# The Role of TNF Receptor-Associated Factor 5 in the Formation of Germinal Centers by B Cells During the Primary Phase of the Immune Response in Mice

**DOI:** 10.3390/ijms252212331

**Published:** 2024-11-17

**Authors:** Mari Hikosaka-Kuniishi, Chieri Iwata, Yusuke Ozawa, Sayaka Ogawara, Tomomi Wakaizumi, Riho Itaya, Ren Sunakawa, Ayaka Sato, Hodaka Nagai, Masashi Morita, Takanori So

**Affiliations:** Laboratory of Molecular Cell Biology, Graduate School of Medicine and Pharmaceutical Sciences, University of Toyama, 2630 Sugitani, Toyama 930-0194, Japan

**Keywords:** Traf5, CD40, germinal center, B cells, antibody, T-dependent immune response, TNF receptor

## Abstract

TNF receptor-associated factors (TRAFs) function as intracellular adaptor proteins utilized by members of the TNF receptor superfamily, such as CD40. Among the TRAF family proteins, TRAF5 has been identified as a potential regulator of CD40. However, it remains unclear whether TRAF5 regulates the generation of germinal center (GC) B cells and antigen-specific antibody production in the T-dependent (TD) immune response. TRAF5-deficient (*Traf5^−/−^*) and TRAF5-sufficient (*Traf5^+/+^*) mice were immunized in the footpad with 2,4,6-trinitrophenol-conjugated keyhole limpet hemocyanin (TNP-KLH) and complete Freund’s adjuvant (CFA). We found that GC B cell generation and antigen-specific IgM and IgG1 production were significantly impaired in *Traf5^−/−^* mice compared to *Traf5^+/+^* mice. The expression levels of CD40-target genes *Fas* and *Lta*, which are involved in GC formation, were significantly decreased in B220^+^ cells isolated from immunized *Traf5^−/−^* mice. *Traf5^−/−^* B cells showed decreased antibody production, proliferation, and induction of CD40-target genes *Tnfaip3*, *Tnfsf4*, and *Cd80* in response to agonistic Fc-CD40L protein in vitro. Furthermore, administration of TNP-KLH and Fc-CD40L to *Traf5^−/−^* mice resulted in a severe loss of GC B cell development. These results highlight the crucial role of TRAF5 in driving CD40-mediated TD immune response in vivo.

## 1. Introduction

The tumor necrosis factor (TNF) receptor-associated factors (TRAFs) are a group of molecules involved in intracellular signaling not only through the TNF receptor (TNFR) and Toll-like receptor superfamilies but also through various unconventional immune receptors such as interleukin (IL) and transforming growth factor (TGF) receptors [1,2,3,4,5,6,7,8,9]. Among the TRAF family proteins, TRAF5 has been identified as a potential regulator of TNFR superfamily members, such as CD40 [10,11,12], and it also modulates signaling through receptors for IL-6 and IL-27 [13,14]. Traf-deficient mice have been generated to investigate the roles of TRAF molecules in health and disease. Although TRAF5 is molecularly and functionally related to TRAF2 and TRAF3, the phenotypes of mice deficient in these TRAF molecules differ significantly. While Traf2- and Traf3-deficient mice exhibit premature death and developmental abnormalities [15,16,17], Traf5-deficient (*Traf5*^−/−^) mice remain healthy with no apparent abnormalities [12]. Unlike TRAF2, TRAF5 has a minor role in lymphoid tissue development [12,18]. These findings suggest that TRAF5 has unique regulatory functions distinct from TRAF2 and TRAF3 [2,19].

It has been proposed that TRAF5 plays a dominant role in B cells due to its relatively high expression levels in this cell population [13,20]. CD40, expressed on B cells, plays a crucial role in germinal center (GC) B cell responses and T-dependent (TD) antibody responses [21,22,23,24]. B cells from *Traf5*^−/−^ mice exhibit impaired proliferation, decreased expression of CD23, CD54, CD80, CD86, and Fas, and secrete lower amounts of immunoglobulin M (IgM) and IgG after in vitro CD40 stimulation [12]. CD40 contains two cytoplasmic TRAF-binding peptide motifs: the TRAF2/3/5-binding motif and the TRAF6-binding motif. TRAF5 is thought to associate with CD40 through TRAF3 [25,26]. Mutations in the TRAF2/3/5-binding motif inhibit GC development and decrease antibody production [27,28,29].

TRAF5 likely plays a significant role in the regulation of GC B cells and TD humoral immune responses mediated by the CD40–CD40 ligand (CD40L) interaction. CD40 expressed on B cells receives signals from T cell-expressed CD40L within the GCs, where antibody diversification and affinity maturation occur [30,31]. Mice deficient in CD40 or CD40L fail to form GC structures or undergo class-switch recombination [22,23,24,32]. Furthermore, activation of the CD40 signaling pathway promotes memory B cell differentiation and antibody production [33,34]. Thus, TRAF5 may regulate GC B cell formation and antigen-specific antibody production in the TD immune response.

In this study, to investigate the role of TRAF5 in the TD immune response mediated by CD40, *Traf5^−/−^* and TRAF5-sufficient (*Traf5^+/+^*) mice were immunized with 2,4,6-trinitrophenol-conjugated keyhole limpet hemocyanin (TNP-KLH) combined with complete Freund’s adjuvant (CFA). We found that antigen-specific IgM and IgG1 levels were significantly lower in *Traf5^−/−^* mice than in *Traf5^+/+^* mice. Moreover, GC B cell generation was significantly reduced in the secondary lymphoid organs of *Traf5^−/−^* mice. When TNP-KLH combined with a CD40L agonist protein was administered to *Traf5^−/−^* mice, GC B cell generation was significantly decreased compared to *Traf5^+/+^* mice. Our findings demonstrate for the first time that TRAF5 is crucial for GC B cell formation during the primary phase of TD immune responses.

## 2. Results

### 2.1. Reduced Antibody Response in Traf5^−/−^ Mice

**Figure 1:**
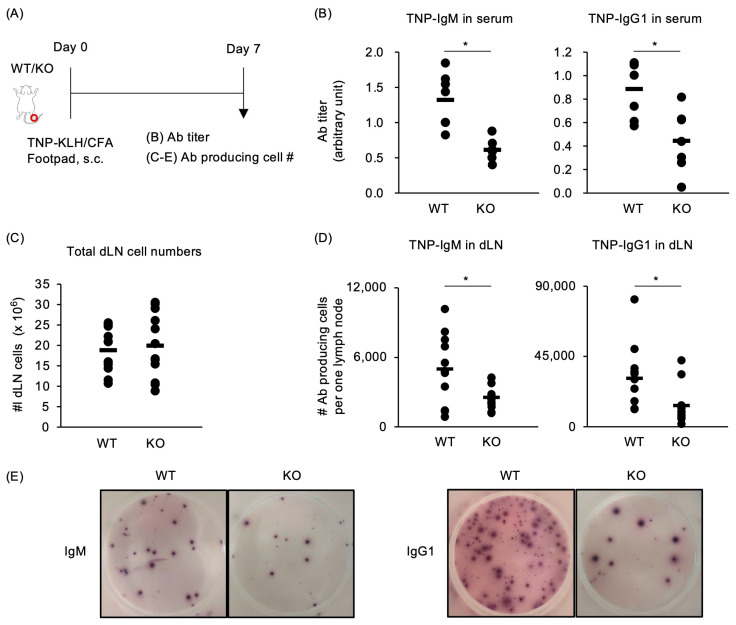
*Traf5^−/−^* mice immunized with TNP-KLH/CFA exhibit attenuated antigen-specific antibody responses. (**A**) Experimental schema. (**B**) TNP-specific IgM and IgG1 antibody (Ab) responses on day 7 in sera from *Traf5^+/+^* (WT) (*n* = 7) and *Traf5^−/−^* (KO) (*n* = 7) mice immunized with trinitrophenol-conjugated keyhole limpet hemocyanin (TNP-KLH) and complete Freund’s adjuvant (CFA) in the footpads, determined by ELISA. (**C**–**E**) Enumeration of TNP-specific IgM- and IgG1-producing cells on day 7 in popliteal draining lymph node (dLN) cells from WT (*n* = 11) and KO (*n* = 12) mice, as shown in B, determined by ELISPOT. Total numbers of dLN cells (**C**) and numbers of anti-TNP IgM- and IgG-producing cells in one dLN (**D**). Representative images of ELISPOT wells (**E**). The bars indicate average values for individual mice. * *p* < 0.05 (Student’s *t*-test).

The role of TRAF5 in TD humoral immune responses remains incompletely understood. To assess whether TRAF5 deficiency affects antigen-specific antibody production in vivo, we immunized *Traf5^+/+^* and *Traf5^−/−^* mice with TNP-KLH combined with CFA in the footpads (Figure 1A). TNP-specific IgM and IgG1 antibodies were detected in the serum of *Traf5^+/+^* mice on day 7. We observed that TNP-specific IgM and IgG1 levels were significantly decreased in the serum of *Traf5^−/−^* mice compared to *Traf5^+/+^* mice (Figure 1B). In unimmunized *Traf5^+/+^* and *Traf5^−/−^* mice, serum levels of anti-TNP IgM and IgG1 were comparable (Figure S1A), indicating that the observed decrease in antibody production in *Traf5^−/−^* mice was due to the immunization procedure. Similarly, the dLNs of immunized *Traf5^−/−^* mice contained significantly fewer anti-TNP IgM and IgG1-producing cells than those of *Traf5^+/+^* mice, even though the total draining lymph node (dLN) cell counts were similar between the groups (Figure 1C–E). These findings suggest that TRAF5 is required for antigen-specific antibody production following antigen administration.

### 2.2. Decreased Germinal Center B Cell Response in Traf5^−/−^ Mice

**Figure 2:**
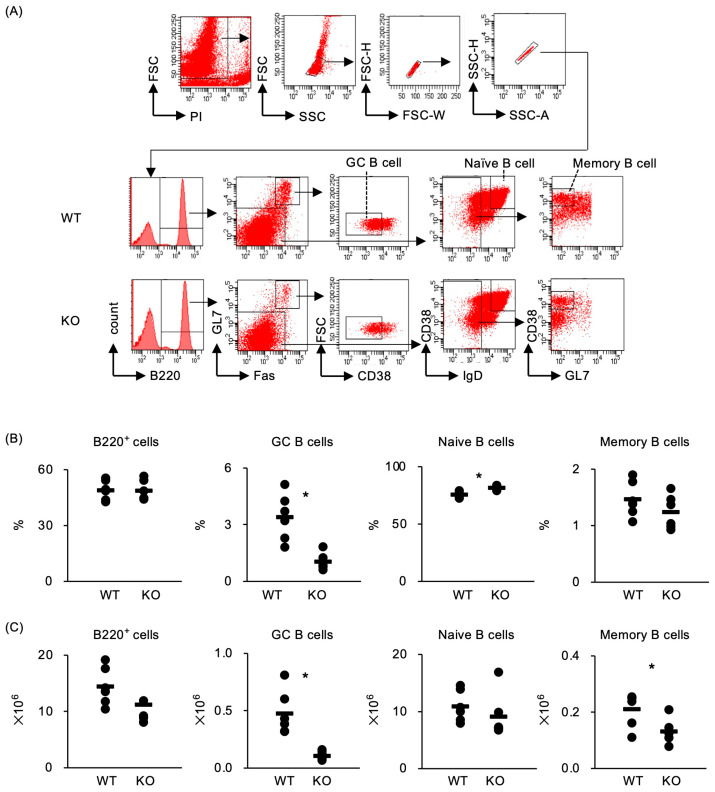
*Traf5^−/−^* mice immunized with TNP-KLH/CFA show decreased percentages of GC B cells. The development of germinal center (GC) B cells, naïve B cells, and memory B cells in WT (*n* = 6) and KO (*n* = 6) mice, as shown in Figure 1, was evaluated by flow cytometry. (**A**) Gating strategy for identifying propidium iodide (PI)-negative live B cell populations: GC B cells (B220^+^Fas^hi^GL7^hi^CD38^low^), naïve B cells (B220^+^IgD^hi^CD38^+^GL7^low^), and memory B cells (B220^+^IgD^low^CD38^+^GL7^low^) in dLN cells of WT and KO mice. Percentages (**B**) and numbers (**C**) of B220^+^ cells, GC B cells, naïve B cells, and memory B cells. The bars indicate average values for individual mice. * *p* < 0.05 (Student’s *t*-test).

The GC reaction plays a central role in TD humoral immunity to antigens. To investigate whether GC formation is affected by TRAF5 deficiency, we assessed the percentages and numbers of GC B cells (B220^+^GL7^hi^FAS^hi^CD38^low^), naïve B cells (B220^+^IgD^hi^CD38^+^GL7^low^), and memory B cells (B220^+^IgD^low^CD38^+^GL7^low^) in immunized *Traf5*^+/+^ and *Traf5^−/−^* mice. Both groups of mice were immunized with TNP-KLH and CFA in one footpad, while PBS was injected into the opposite footpad as a control. GC B cells, naïve B cells, and memory B cells were detected in the dLNs from the TNP-KLH-immunized footpad in both groups. Importantly, the dLNs of *Traf5^−/−^* mice contained significantly lower percentages and numbers of GC B cells compared to those of *Traf5^+/+^* mice (Figure 2A–C). In the dLNs from the PBS-injected footpad, GC B cells were barely detected, with comparable percentages and numbers between groups (Figure S1B,C). These results indicate that TRAF5 promotes the generation of GC B cells under immunized conditions. Although GC formation is detectable in Peyer’s patches (PPs) even in the steady state [35], the percentages of GC B cells in PPs did not show a statistically significant difference between groups (Figure S2A,B). Furthermore, the numbers of IgA- and IgM-producing cells in PPs were not significantly different between the groups (Figure S2C). These data demonstrate that TRAF5 deficiency impairs the generation of GC B cells and antigen-specific antibody production during the primary phase of the TD immune response.

### 2.3. Defective Induction of CD40-Target Genes by B Cells in Traf5^−/−^ Mice

**Figure 3:**
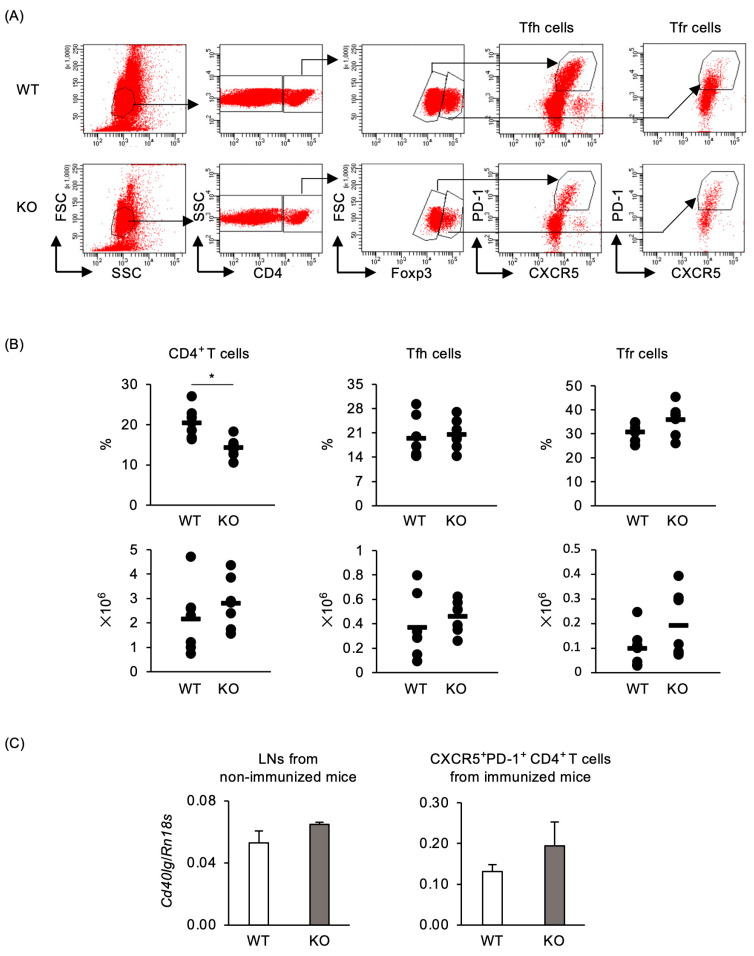
*Traf5^−/−^* mice immunized with TNP-KLH/CFA exhibit normal development of Tfh and Tfr cells. The development of follicular helper T (Tfh) and follicular regulatory T (Tfr) cells in WT (*n* = 7) and KO (*n* = 7) mice, as shown in Figure 1, was evaluated by flow cytometry. (**A**) Gating strategy for identifying Foxp3^−^PD-1^+^CXCR5^+^CD4^+^ Tfh cells and Foxp3^+^PD-1^+^CXCR5^+^CD4^+^ Tfr cells in dLN cells from WT and KO mice. (**B**) Percentages and numbers of Tfh and Tfr cell populations. Bars indicate average values for individual mice. (**C**) Expression levels of *Cd40lg* in popliteal LNs from non-immunized mice and in CXCR5^+^PD-1^+^CD4^+^ T cells from popliteal LNs of mice 7 days post-immunization, as shown in Figure 1. Data are presented relative to *Rn18s* expression as mean ± standard deviation (*n* = 3), from a representative experiment of two independent experiments showing similar results. * *p* < 0.05 (Student’s *t*-test).

The mechanisms by which TRAF5 regulates GC B cells are not well understood. Follicular helper T cells (Tfh) and follicular regulatory T cells (Tfr) are known to regulate GC formation and maintenance [30,36,37]. We hypothesized that a decrease in Tfh cells or an increase in Tfr cells due to TRAF5 deficiency might lead to the reduction in GC B cells. To test this hypothesis, we measured Tfh and Tfr cell populations in the dLNs of immunized mice by flow cytometry (Figure 3A). However, the percentages and numbers of Tfh and Tfr cells were not significantly different between the groups (Figure 3B).

Next, we investigated whether reduced CD40L expression in *Traf5*^−/−^ T cells might lead to a decrease in GC B cell development. CD40L expression is detectable in non-immunized LNs [38]. We assessed CD40L expression in non-immunized popliteal LNs and in CXCR5^+^PD-1^+^CD4^+^ T cells, which include Tfh and Tfr cells, in immunized mice (Figure 3C and Figure S3). No significant differences in CD40L expression were observed in non-immunized popliteal LNs or in CXCR5^+^PD-1^+^CD4^+^ T cells between the groups. These findings suggest that the reduced GC B cell response and antibody production in immunized *Traf5^−/−^* mice are not due to alterations in Tfh and Tfr cell activity or CD40L expression.

**Figure 4:**
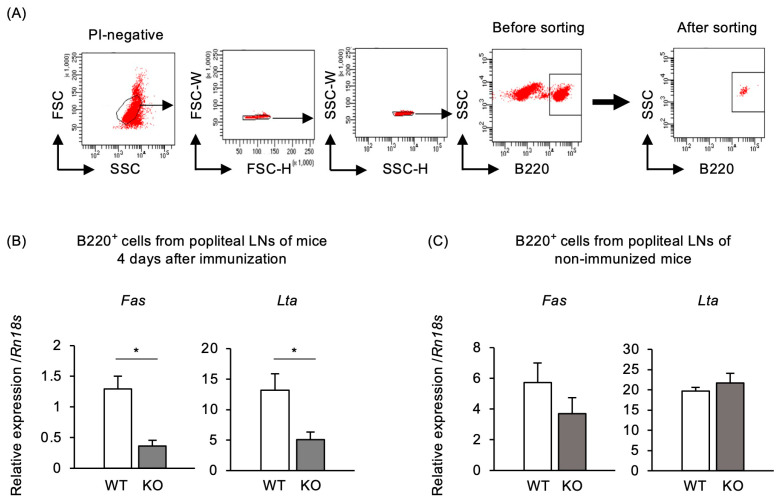
B cells from *Traf5^−/−^* mice immunized with TNP-KLH/CFA show reduced expression of CD40-target genes. Expression levels of CD40-target genes, *Fas* and *Lta*, in B cells from WT and KO mice were evaluated using real-time RT-PCR. (**A**) Representative gating strategy for sorted B220^+^ B cell populations from the popliteal dLN cells of WT and KO mice. (**B**) Expression levels of *Fas* and *Lta* in sorted B220^+^ cells from the popliteal dLN cells of WT and KO mice, 4 days post-immunization. (**C**) Expression levels of *Fas* and *Lta* in sorted B220^+^ cells from unimmunized WT and KO mice. Data are presented relative to *Rn18s* expression as mean ± standard deviation (*n* = 3) from a representative experiment of two independent experiments showing similar results. * *p* < 0.05 (Student’s *t*-test).

TRAF5 deficiency may lead to defective GC B cell and TD humoral responses due to impaired CD40 signaling. The interaction between CD40, expressed by B cells, and CD40L, expressed by activated CD4^+^ T cells, is essential for GC responses. This interaction induces upregulation of lymphotoxin-alpha (Lta) and Fas in B cells, with Lta expression playing a key role in GC formation [39,40,41,42]. Thus, we investigated whether TRAF5 deficiency is associated with reduced expression of *Lta* and *Fas* in B220^+^ B cells from the dLNs 4 days after immunization. The expression levels of these genes were indeed significantly lower in B cells from the dLNs of TNP-KLH-immunized *Traf5^−/−^* mice than in those from *Traf5^+/+^* mice (Figure 4A,B). In contrast, gene expression levels in the dLNs from PBS-injected footpads were similar between groups (Figure 4C). These findings suggest that TRAF5 deficiency reduces CD40 signaling in B cells during the primary antigen-specific immune response. This reduction in *Fas* and *Lta* expression in B220^+^ cells 4 days post-immunization in *Traf5^−/−^* mice likely leads to impaired GC B cell formation observed on day 7 post-immunization (Figure 2).

### 2.4. Decreased Responsiveness of Traf5^−/−^ B Cells to CD40L In Vitro

It remains unclear whether TRAF5 deficiency affects the generation of GC B cells mediated by CD40 in vivo. To analyze the role of TRAF5 in CD40 signaling, we prepared a recombinant CD40L protein, Fc-CD40L (Figure S4). Fc-CD40L contains the human IgG1 Fc region, a PA tag, the collagen-like domain from mannose-binding lectin 1 (MBL), the extracellular region of mouse CD40L, and a His tag. The Fc and collagen-like domains were used to facilitate the formation of oligomeric trimer structures of CD40L. We confirmed that Fc-CD40L specifically bound to CD40-Fc (Figure S5A) and displayed dimer and multimer structures in SDS-PAGE under non-boiled and non-reduced conditions ((−), Figure S5B). This result is consistent with a previous report from our laboratory, which shows that MBL-containing structures are more likely to form active oligomers [43].

*Traf5*^+/+^ and *Traf5*^−/−^ B cells express equivalent levels of CD40 on their surface (Figure S5C), and Fc-CD40L similarly bound to both *Traf5^+/+^* and *Traf5^−/−^* B cells in a dose-dependent manner (Figure S5D). Naïve B cells from *Traf5^+/+^* mice proliferated in a dose-dependent manner upon stimulation with Fc-CD40L and IL-4 (Figure S5E). These results demonstrate that Fc-CD40L functions as an agonist and is useful for in vitro and in vivo assays to evaluate CD40 activity.

**Figure 5:**
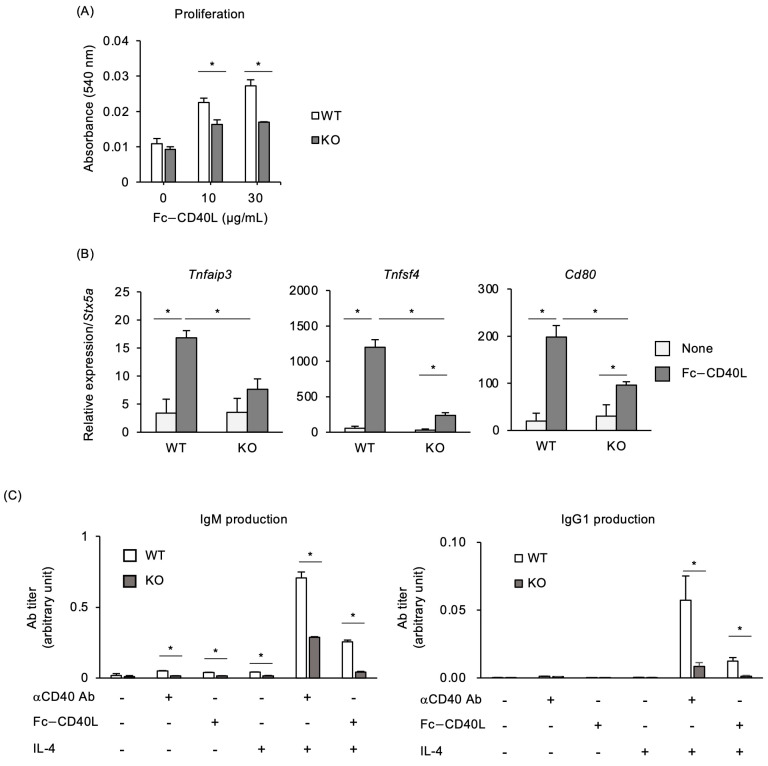
*Traf5^−/−^* B cells are refractory to CD40L stimulation in vitro. CD40L-mediated cell proliferation (**A**), gene expression (**B**), and IgM and IgG1 production (**C**) in B cells from unimmunized WT and KO mice were assessed using MTT assay, real-time RT-PCR, and ELISA, respectively. (**A**) Proliferative responses of splenic naïve B cells cultured with the indicated concentrations of Fc-CD40L for 3 days. (**B**) Expression levels of CD40L-target genes, *Tnfaip3*, *Tnfsf4*, and *Cd80*, in B cells treated with 30 µg/mL Fc-CD40L for 7 h. Data are normalized to *Stx5a* expression and presented as mean ± standard deviation (*n* = 3) from a representative experiment of two independent experiments showing similar results. (**C**) IgM and IgG1 production of splenic naïve B cells cultured with combinations of Fc-CD40L (10 µg/mL), anti-CD40 Ab (4 µg/mL), and IL-4 (10 ng/mL) for 4 days, evaluated by ELISA. * *p* < 0.05 ((**A**,**C**): Student’s *t*-test; (**B**): one-way ANOVA with Tukey’s post hoc test).

Importantly, using Fc-CD40L, we confirmed that proliferation was significantly decreased in *Traf5^−/−^* B cells compared to *Traf5^+/+^* B cells in vitro (Figure 5A). Additionally, Fc-CD40L significantly upregulated the CD40-target genes *Tnfaip3*, *Tnfsf4*, and *Cd80* in B cells. However, the expression levels of these genes were significantly lower in *Traf5^−/−^* B cells than in *Traf5^+/+^* B cells in vitro (Figure 5B). An increase in IgM and IgG1 production was observed in *Traf5^+/+^* B cells upon stimulation with Fc-CD40L and IL-4, as well as with anti-CD40 agonistic antibody and IL-4 (Figure 5C). Under these conditions, IgM and IgG1 produced by *Traf5^−/−^* B cells were significantly lower than those produced by *Traf5^+/+^* B cells. These findings indicate that TRAF5 promotes CD40 signaling, which is required for cell proliferation, activation, and antibody production, and suggest that TRAF5 signaling downstream of CD40 supports the induction of the GC response.

### 2.5. Impaired Generation of GC B Cells in Traf5^−/−^ Mice upon Exposure to Antigen and CD40L

**Figure 6:**
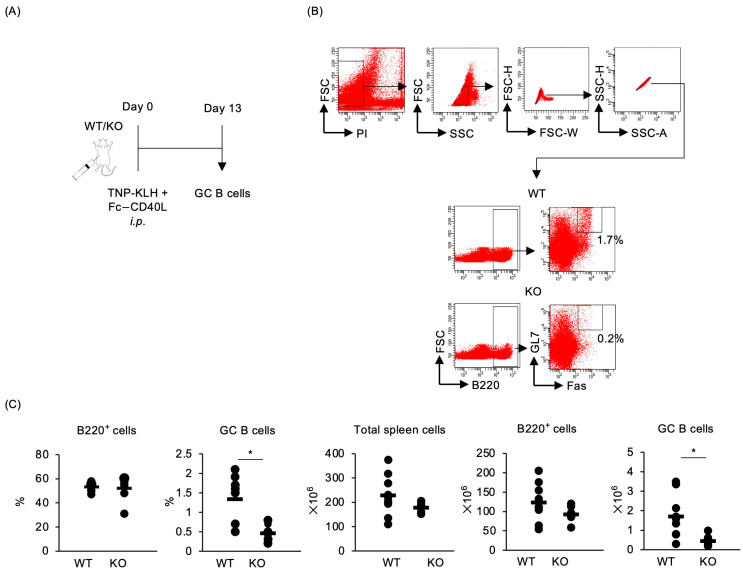
*Traf5^−/−^* mice primed with TNP-KLH and CD40L display defective development of GC B cells. The development of GC B cells in WT (*n* = 9) and KO (*n* = 7) mice was evaluated by flow cytometry. (**A**) Experimental schema. (**B**) Gating strategy for identifying PI-negative, Fas^+^GL7^+^B220^+^ GC B cells in the splenocytes of WT and KO mice immunized intraperitoneally with TNP-KLH and Fc-CD40L. (**C**) Percentages and numbers of total spleen cells, B220^+^ cells, and GC B cells in WT and KO mice, 13 days post-immunization. Bars represent average values for individual mice. * *p* < 0.05 (Student’s *t*-test).

To examine whether TRAF5 deficiency affects the generation of GC B cells via CD40 in vivo, *Traf5^+/+^* and *Traf5^−/−^* mice were intraperitoneally administered TNP-KLH as an antigen and Fc-CD40L as an adjuvant on day 0 (Figure 6A). The generation of GC B cells was assessed in the spleens of immunized mice on day 13 (Figure 6B). Interestingly, the percentages and numbers of GC B cells were significantly decreased in *Traf5^−/−^* mice compared to *Traf5^+/+^* mice (Figure 6B,C). *Traf5*^−/−^ mice exhibited a decreasing tendency in serum TNP-specific IgM and IgG1 levels (Figure S6). These data demonstrate that TRAF5 is required for CD40 signaling, which is essential for the GC B cell response.

## 3. Discussion

In this study, we investigated whether TRAF5 deficiency affects antigen-specific antibody production and GC formation via CD40 in TD immune responses. We found that the generation of GC B cells, as well as the induction of antigen-specific IgM and IgG1, was significantly reduced in immunized *Traf5^−/−^* mice. In vitro experiments showed that *Traf5^−/−^* B cells exhibited attenuated antibody production, reduced proliferation, and decreased expression of CD40-target genes. Furthermore, the administration of Fc-CD40L was unable to promote GC B cell generation in *Traf5^−/−^* mice. These data demonstrate that TRAF5 critically contributes to CD40-mediated TD immune responses in vivo.

The distinct phenotypes of *Traf5^+/+^* and *Traf5^−/−^* mice in antibody responses may depend on the adjuvants used in the experiments. We observed impaired antigen-specific IgM and IgG1 responses in *Traf5^−/−^* mice immunized with TNP-KLH and CFA (Figure 1), suggesting a critical involvement of TRAF5 in CD40 signaling. Immunization of *Traf5^+/+^* and *Traf5^−/−^* mice with 4-hydroxy-3-nitrophenyl-acetyl-chicken gamma globulin (NP-CGG) and alum adjuvant did not result in a significant decrease in antigen-specific antibody production in *Traf5*^−/−^ mice [12]. Agonistic anti-CD40 antibodies and recombinant CD40L proteins exhibit broad immunostimulatory effects, and thus, these reagents can be used as adjuvants [44,45,46,47,48]. Immunization of mice with TNP-KLH and Fc-CD40L could induce anti-TNP IgM and IgG1 responses; however, we did not observe a statistically significant difference between the groups (Figure S6). The inflammatory cytokine milieu induced by CFA may facilitate antigen-specific interactions between CD40L-expressing CD4^+^ T cells and CD40-expressing B cells in secondary lymphoid organs, leading to optimal antibody production.

We found for the first time that *Traf5^−/−^* mice exhibited significantly defective formation of GC B cells after immunization with TNP-KLH/CFA or TNP-KLH/Fc-CD40L (Figure 2 and Figure 6). CD40L-deficient mice exhibit impaired GC formation [23,24]. B cells interacting with T cells differentiate into early GC B cells around 4 days post-immunization [49]. At day 4, the expression levels of the CD40 target genes *Lta* and *Fas* in activated B cells were significantly lower in *Traf5*^−/−^ mice compared with *Traf5*^+/+^ mice (Figure 4), suggesting that TRAF5 deficiency suppresses CD40 activation, preventing B cells from differentiating into GC B cells. In addition, *Traf5^+/+^* and *Traf5^−/−^* mice had comparable proportions of Tfh and Tfr cells and similar levels of CD40L expression (Figure 3). These data suggest that the reduction in GC B cell formation in *Traf5^−/−^* mice is due to an intrinsic defect in CD40 signaling in B cells rather than to indirect effects via Tfh or Tfr cells.

*Traf5^−/−^* B cells showed significantly defective responses to CD40L stimulation, including attenuated antibody production, decreased proliferation, and reduced expression of CD40-target genes, *Cd80*, *Tnfsf4*, and *Tnfaip3* (Figure 5). Our previous study also indicated that CD40-induced genes, such as *Icam1*, *Fas*, and *Lta*, were significantly decreased in *Traf5^−/−^* B cells after stimulation with anti-CD40 agonistic antibody [14]. Nakano et al. previously showed that the surface upregulation of Cd23, Cd54, Cd80, Cd86, and Fas was reduced in *Traf5^−/−^* B cells after stimulation with anti-CD40 agonistic antibody. IgM and IgG1 production and B cell proliferation induced by anti-CD40 agonistic antibody or CD40L protein were substantially decreased in *Traf5^−/−^* B cells [12]. These results consistently demonstrate that TRAF5 functions as a mediator and component of CD40 signaling.

It is unclear how the cytoplasmic domain of CD40 regulates the signaling activities mediated by TRAF2, TRAF3, and TRAF5 in terms of GC reactions and antibody responses. TD humoral immunity, which includes CD40-mediated antibody isotype switching and GC B cell formation, is inhibited in B cell-intrinsic Traf2 deficiency but not in Traf3 deficiency [50,51]. TRAF2 and TRAF5 play redundant roles in TNFR1 signaling [52,53]. Thus, in the context of GC reactions and antibody production in TD immune responses, TRAF5 and TRAF2 may work together to control CD40 signaling. Further analysis will be important to determine how TRAF5 cooperates with other TRAF family molecules to regulate CD40-mediated GC B cell and antibody responses.

TRAF5 has been reported to function as a negative regulator of TLR signaling in B cells [54]. *Traf5^−/−^* B cells produce increased levels of pro-inflammatory cytokines in response to TLR ligands in vitro. TRAF5 appears to associate with TAB2 following TLR ligation and inhibits MAPK signaling. Thus, TRAF5 functions differently depending on its receptor partners. Consequently, further research is required to examine how TRAF5 modulates the immune response via its interacting immune receptors in vivo.

In summary, our study demonstrates that TRAF5 mediates CD40 signaling to induce GC formation in the TD immune response to antigens. TRAF5’s roles in immune receptors have been suggested to be involved in inflammatory and autoimmune diseases in humans [1,2,19,55]. In particular, mutations in the TRAF5 gene are associated with rheumatoid arthritis [56], ankylosing spondylitis [57], and uveitis [58,59]. A decrease in TRAF5 expression in B cells is linked to the development of autoreactive B cells that are responsible for systemic lupus erythematosus [60]. Further research will be essential to elucidate the consequences of TRAF5 deficiency in terms of dysregulation of adaptive immunity and to clarify the specific and redundant roles of TRAF5 in lymphocyte responses in health and disease.

## 4. Materials and Methods

### 4.1. Mice

*Traf5^+/+^* and *Traf5^−/−^* mice were generated by crossing *Traf5^−/−^* mice with C57BL/6 mice. The offspring of heterozygous *Traf5^+/-^* mice, which had been backcrossed with C57BL/6 mice more than ten times, were intercrossed to produce *Traf5^+/+^* and *Traf5^−/−^* mice for this experiment [12,13]. The animals were bred and housed in a room set to 24 ± 3 °C with a 12 h light/dark cycle (lights on at 7:00 AM) and had ad libitum access to food and water under specific pathogen-free conditions at the Life Science Research Center, University of Toyama. Animal experimental protocols were approved by the Animal Care and Use Committee of the University of Toyama (Approval Numbers: A2024PHA-02 and A2024PHA-03) and were conducted in accordance with the Institutional Animal Experiment Handling Rules of the University of Toyama. Both male and female mice (6–12 per experiment) were used in the in vivo studies. The animals were employed in experiments for up to 14 days after immunization. At the end of the experiments, the mice were euthanized via cervical dislocation.

### 4.2. Antibodies

Antibodies used in this study are as follows: phycoerythrin (PE)/Cyanine7 (PECy7)-conjugated anti-mouse CD4 (100421, BioLegend, San Diego, CA, USA), PE-conjugated anti-mouse CD185 (CXCR5) (145503, BioLegend), allophycocyanin (APC)-conjugated anti-mouse CD279 (PD-1) (135209, BioLegend), Pacific Blue-conjugated anti-mouse/human CD45R/B220 (103230, BioLegend), Alexa Fluor^®^ 488-conjugated anti-mouse/human CD45R/B220 (103228, BioLegend), Pacific Blue-conjugated anti-mouse IgD (405711, BioLegend), Alexa Fluor^®^ 647-conjugated anti-mouse/human GL7 (144606, BioLegend), PE-conjugated anti-mouse CD95 (Fas) (152607, BioLegend), PECy7-conjugated anti-mouse CD38 (102717, BioLegend), biotin-conjugated anti-mouse Foxp3 (13-5773-80, Thermo Fisher Scientific, Waltham, MA, USA), biotin-conjugated anti-mouse IgG2b (406703, BioLegend), fluorescein isothiocyanate (FITC)-conjugated streptavidin (405201, BioLegend), alkaline phosphatase (AP)-conjugated goat anti-mouse IgM (1020-04, SouthernBiotech, Birmingham, AL, USA), AP-conjugated goat anti-mouse IgG1 (1070-04, SouthernBiotech), PECy7-conjugated anti-mouse CD45R/B220 (103221, BioLegend), FITC-conjugated anti-mouse CD40 (102905, BioLegend), Ultra-LEAF™ purified anti-mouse CD40 (102811, BioLegend), biotin-conjugated anti-human IgG1 antibody (109-065-190, Jackson ImmunoResearch Laboratories, Inc., West Grove, PA, USA), and streptavidin-PE (405203, BioLegend).

### 4.3. Fc−CD40L and CD40−Fc Proteins

PCR primers were purchased from Fasmac Co., Ltd. (Kanagawa, Japan). A vector containing cDNA encoding a PA peptide (GVAMPGAEDDVV; ggc gtt gcc atg cca ggt gcc gaa gat gat gtg gtg) tag, the collagen-like domain of mouse mannose-binding lectin 1 (^18^Ser−^126^Gly; *Mbl1*, NM_010775), and the extracellular domain of mouse OX40 ligand has been previously described [43]. The recombinant OX40 ligand protein, which includes the collagen-like domain and the PA tag, assembles into a trimeric structure and binds to either the cell surface OX40 receptor or the OX40-Fc fusion protein. Based on the cDNA sequences of mouse *Cd40l* (*Tnfsf5*, NM_011616) and mouse *Cd40* (*Tnfrsf5*, M83312), the cDNAs of the entire coding regions were amplified by PCR using specific primers and PrimeSTAR^TM^ GXL DNA polymerase (R050A, Takara Bio, Shiga, Japan), and the resulting PCR fragments were inserted into the vector Zero Blunt^TM^-TOPO^TM^ PCR Cloning Kit (450245, Thermo Fisher Scientific).

To construct a fusion gene for PA−MBL−CD40L−His, genes encoding the PA-peptide tag−collagen-like domain of Mbl1 (PA−MBL), the extracellular domain of Cd40l (^114^R-^260^L), and the His_6_-peptide (HHHHHH; cat cat cac cat cac cat) were amplified and combined using recombinant PCR before being inserted into the TOPO vector. Using In-Fusion cloning (638947, In-Fusion Snap Assembly Master Mix, Takara Bio), the signal sequence of the human interleukin-2 receptor gamma chain (hIL-2Rγ signal sequence, LKPSLPFTSLLFLQLPLLGVGLNTTILTP, BAA03861.1, NM_000206.3) was inserted into the N-terminus of the Fc region (hinge, CH_2_, and CH_3_ domains) of human IgG1 genomic DNA (Homo sapiens recombinant IgG1 heavy chain gene, AF237583.1) in the pEF−Fc expression vector [61], which is derived from the pEF-BOS vector [62]. The PA−MBL−CD40L−His gene was inserted into the C-terminus of the Fc region of human IgG1 genomic DNA. The entire hIL-2Rγ signal sequence−Fc−PA−MBL−CD40L−His_6_ gene was amplified by PCR. Primers were used that added a 5′ EcoRI site in the forward primer and a 3′ BglII site in the reverse primer. The resulting PCR fragment was digested with EcoRI (R3101, New England Biolabs, Ipswich, MA, USA) and BglII (R0144, New England Biolabs), followed by ligation into the mammalian expression vector pCAGGS (LT727518.1) [63] (Figure S3).

A pCAGGS vector encoding a fusion gene that consists of the mouse Cd40 extracellular region (^24^Val-^193^Arg) at the N-terminus of human IgG1-Fc (CD40-Fc) was constructed. A PCR fragment (gac tac aag gat gac gat gac aag ctc gat gga gga tac cca tac gat gtt cca gat tac gct), which corresponds to a Flag−HA tag peptide (DYKDDDDKLDGGYPYDVPDYA), was inserted into the 3′ end of the CD40−Fc gene, using In-Fusion cloning (Figure S3).

To produce Fc−CD40L and CD40−Fc recombinant proteins, the expression vector was transfected into HEK293T cells (CRL-3216, ATCC, Manassas, VA, USA) using polyethyleneimine (PEI) (408727, Merck, Burlington, MA, USA). The cells were cultured in DMEM (043-30085, FUJIFILM Wako Pure Chemical Corporation, Osaka, Japan) supplemented with 2% fetal calf serum (FCS), 100 U/mL penicillin, and 100 µg/mL streptomycin for 4 days. The medium containing the recombinant protein was then applied to a HiTrap^TM^ rProtein A FF column (17507901, Cytiva, Tokyo, Japan). After rinsing the column with phosphate-buffered saline (PBS), the bound Fc protein was eluted with an elution buffer (80 mM glycine-HCl, 0.8 M arginine, pH 3.0), and the protein fractions were immediately neutralized with 1 M Tris-HCl (pH 9.0). The eluted protein was dialyzed against PBS and filtered through a Millex-GP (0.22 µm, SLGPR33RS, Merck) filter. The total protein concentration was determined using a bicinchoninic acid (BCA) assay (297-73101, FUJIFILM Wako). Fc−CD40L and CD40−Fc proteins were mixed with 4× Laemmli sample buffer (240 mM Tris–HCl, pH 6.8, 40% glycerol, 8% sodium dodecyl sulfate (SDS), and 0.1% bromophenol blue) and separated by SDS-polyacrylamide gel electrophoresis (PAGE), followed by Coomassie blue staining (Ezstain Aqua, AE-1340, ATTO Corp., Tokyo, Japan).

### 4.4. B Cells

Resting B cells were isolated from the spleens of *Traf5*^+/+^ and *Traf5*^−/−^ mice using CD43 (Ly48) microbeads (130-049-801, Miltenyi Biotec, Bergisch Gladbach, Germany). The cells were cultured in RPMI 1640 medium supplemented with 10% heat-inactivated fetal calf serum (FCS), 100 U/mL penicillin, 100 µg/mL streptomycin, 2 mM L-alanyl-L-glutamine, and 50 µM 2-mercaptoethanol (2-ME). Cell proliferation was assessed using a 3-(4,5-dimethylthiazol-2-yl)-2,5-diphenyl tetrazolium bromide (MTT) assay (M2128, Merck). B cells (2.5–5 × 10^5^ cells/mL) were plated in 96-well round-bottom culture plates and stimulated with Fc-CD40L, with or without recombinant mouse IL-4 (10 ng/mL, 214-14, PeproTech, Inc., Cranbury, NJ, USA) for 3 days. To assess the expression of CD40-target genes, B cells (5 × 10^6^ cells/mL) were stimulated with Fc-CD40L in 96-well flat-bottom plates for 7 h. For the detection of IgM and IgG1 production, B cells were stimulated with combinations of Fc-CD40L (10 µg/mL), anti-CD40 Abs (4 µg/mL, 102811, BioLegend), and IL-4 (10 ng/mL, 214-14, PeproTech) in 96-well round-bottom plates for 4 days.

### 4.5. Antigen

KLH (74805-250MG, Merck) was dissolved in 3 mL of borate buffered saline (BBS) (0.17 M Borate, 0.12 M NaCl, pH 8.0) at a concentration of 20 mg/mL and stirred overnight at 4 °C. Picrylsulfonic acid (T1340, Tokyo Chemical Industry Co., Ltd., Tokyo, Japan), dissolved in BBS, was added to the KLH solution (38 µg picrylsulfonic acid per 90 mg KLH) and stirred overnight in the dark at 4 °C. The reaction mixture was applied to a PD-10 column (17-0851-01, Cytiva, Tokyo, Japan), which was then eluted with PBS. The void fraction was collected. The extent of TNP modification in KLH was determined by measuring absorbance at 280 nm and 340 nm [64] using a NanoDrop 2000 (ND-2000, Thermo Fisher Scientific). TNP-KLH, with approximately 10 to 20 molecules of TNP per molecule of KLH, was prepared in our laboratory.

### 4.6. Immunization

*Traf5*^+/+^ and *Traf5*^−/−^ mice (3-4 per group), including both male and female mice, were subcutaneously immunized in the footpad with 50–60 μL of 100 µg TNP_20_-KLH mixed with CFA (F5881, Merck). Control mice were injected with PBS in the footpad as a control. Seven days after immunization, flow cytometry and enzyme-linked immunospot (ELISPOT) assays were performed using the popliteal LNs as dLNs. TNP-specific IgM and IgG1 levels were measured using an enzyme-linked immunosorbent assay (ELISA). CXCR5^+^PD-1^+^CD4^+^ T cells were positively isolated from immunized mice using a FACS Aria SORP (BD Biosciences, Franklin Lakes, NJ, USA). The sorted CXCR5^+^PD-1^+^CD4^+^ T cells and popliteal LNs from non-immunized mice were used for the detection of CD40L mRNA by qPCR. Four days after immunization, B220^+^ cells were sorted using a FACS Aria SORP (BD Biosciences). For the administration of Fc-CD40L, *Traf5*^+/+^ and *Traf5*^−/−^ mice were immunized intraperitoneally with TNP_12_-KLH (100 μg/mouse) and Fc-CD40L (100 μg/mouse). GC B cells were detected on day 13, and TNP-specific IgM and IgG1 levels were measured on day 11 after immunization.

### 4.7. Real-Time RT-PCR

Total RNA was extracted from cells using Isogen II (Nippon Gene, Toyama, Japan), and cDNA was synthesized using the ReverTra Ace qPCR RT Master Mix with genomic DNA remover (FSQ-301, TOYOBO, Osaka, Japan). Diluted cDNA was used for quantitative RT-PCR with the Brilliant III Ultra-Fast SYBR qPCR Master Mix (600882, Agilent Technologies, Santa Clara, CA, USA) on a CFX Connect (Bio-Rad Laboratories, Inc., Hercules, CA, USA). The results are presented relative to the abundance of transcripts encoding *Rn18s* (forward primer, 5′-CGTTCTTAGTTGGTGGAGC-3′; reverse primer, 5′-TAAGGGCATCACAGACCT-3′) or *Stx5a* (forward primer, 5′-GCACACATGGTTAAAGAACAGGAG-3′; reverse primer, 5′-CAGGCAAGGAAGACCACAAAG-3′). The primers used were as follows: *Fas* (forward primer, 5′-GCGGGTTCGTGAAACTGATAA-3′; reverse primer, 5′-GCAAAATGGGCCTCCTTGATA-3′), *Lta* (forward primer, 5′-CCCATCCACTCCCTCAGAAG-3′; reverse primer, 5′-CATGTCGGAGAAAGGCACGAT-3′), *Tnfaip3* (forward primer, 5′-GCCCAGTCTGTAGTCTTCGG-3′; reverse primer, 5′-TTGTTCAGCCATGGTCCTCG-3′), *Tnfsf4* (forward primer, 5′-AATCTGGAAAACGGATCAAGGC-3′; reverse primer, 5′-CAGGCAGACATAGATGAAGCAC-3′), *Cd80* (forward primer, 5′-ACCCCCAACATAACTGAGTCT-3′; reverse primer, 5′-TTCCAACCAAGAGAAGCGAGG-3′), and *Cd40lg* (forward primer, 5′-ACACGTTGTAAG CGAAGCCA-3′, reverse primer, 5′-AATGGGCGTTGACTCGAAGG-3′).

### 4.8. Flow Cytometry

Cells were stained with antibodies for cell surface markers. To remove dead cells, propidium iodide (PI) (341-078881, FUJIFILM Wako) was used. For the staining of intracellular Foxp3, cells were fixed and permeabilized with fixation/permeabilization buffer (00-5123-43, Thermo Fisher Scientific) after staining surface molecules, and then stained with biotin-conjugated anti-mouse Foxp3 and biotin-conjugated anti-mouse IgG2b, followed by streptavidin-FITC. For the analysis of PPs, all PPs were collected from one mouse and digested with 5 mM ethylenediaminetetraacetic acid (EDTA)/Hank’s/10% FCS for 15 min, repeated 2 to 4 times, and the single-cell suspension was stained with the respective antibodies for flow cytometry. The remaining single-cell suspension was used for the ELISPOT assay. For the binding assay of Fc-CD40L to B220^+^ cells, splenocytes were incubated with PECy7-conjugated anti-B220 and Fc-CD40L proteins, followed by incubation with biotin-conjugated anti-human IgG1 antibody (109-065-190, Jackson ImmunoResearch Laboratories, Inc.), and then with streptavidin-PE. Data were acquired on a FACS Canto II or a Celesta (BD Biosciences) and analyzed with BD FACS Diva software version 9.

### 4.9. ELISPOT

To detect TNP-specific IgM- and IgG1-producing cells, we employed a previously described method [65]. Briefly, dLNs from immunized mice (0.5–1 × 10^5^ cells/well) were added to ELISPOT plates (MultiScreen HTS^TM^ MSHAS4510, Merck KGaA, Darmstadt, Germany) pre-coated with 10 μg/mL of TNP-conjugated bovine serum albumin (BSA), provided by Dr. S.-I. Hayashi from Tottori University, Japan. The binding between the TNP antigen and anti-TNP antibodies was visualized using AP-conjugated goat anti-mouse IgM (1020-04, Southern Biotech, Birmingham, AL, USA) and IgG1 (1070-04, Southern Biotech) at a 1:1000 dilution, with Nitroblue Tetrazolium (022-08661, FUJIFILM Wako) and 5-Bromo-4-chloro-3-indolyl-phosphate (148-01991, FUJIFILM Wako) as substrates. After photographing each well, the number of spots was counted. The number of TNP-IgM- and TNP-IgG1-producing cells per lymph node was then calculated by multiplying the total number of lymph node cells by the frequency measured in ELISPOT.

For the detection of IgA- and IgM-producing cells in PPs, unlabeled goat anti-mouse Ig (1010-01, Southern Biotech) was pre-coated onto ELISPOT wells instead of TNP-BSA. After PP cells from non-immunized mice (1 × 10^5^ cells/well) were added to the ELISPOT plates, AP-conjugated goat anti-mouse IgA (1040-04, Southern Biotech) and AP-conjugated goat anti-mouse IgM were used for detection. The number of IgA- and IgM-producing cells per PP was then calculated by multiplying the total number of PP cells by the frequency measured in ELISPOT.

### 4.10. ELISA

To detect TNP-specific IgM and IgG1, mouse serum was added to ELISA plates (439454, Thermo Fisher Scientific) pre-coated with 0.08 μg/mL of TNP-BSA. The binding between the TNP antigen and anti-TNP antibodies was visualized using AP-conjugated goat anti-mouse IgM (1020-04, Southern Biotech) and IgG1 (1070-04, Southern Biotech) at a 1:1000 dilution, along with the Alkaline Phosphatase Yellow (pNPP) Liquid Substrate System for ELISA (P7998-100ML, Merck). Absorbance was measured at 405 nm using a FilterMax F5 (Molecular Devices, San Jose, CA, USA). Serum antibody titers were determined by comparing test samples to a standard curve generated with a representative antiserum assigned a value of one arbitrary unit. To detect IgM and IgG1 in the in vitro assay, goat anti-mouse IgG and human ads-UNLB (1010-01, Southern Biotech) were pre-coated on ELISA plates instead of TNP-BSA. The same method was employed for the subsequent detection conditions. IgM and IgG1 antibody titers were determined by comparing the test samples to a standard curve generated with a representative sample assigned a value of one arbitrary unit. To detect the binding between CD40-Fc and Fc-CD40L, purified Fc-CD40L proteins were added to ELISA plates (439454, Thermo Fisher Scientific) pre-coated with CD40-Fc. Fc-CD40L was detected using anti-PA tag IgG (NZ-1, 012-25863, FUJIFILM Wako) and peroxidase-conjugated AffiniPure Goat Anti-Rat IgG (H+L) (112-035-28167, Jackson ImmunoResearch). Absorbance was measured at 450 nm using a FilterMax F5.

### 4.11. Statistical Analysis

Statistical significance was evaluated using either a one-way analysis of variance with Tukey’s post-hoc test (EZR software version 1.36, Saitama Medical Center, Jichi Medical University, Saitama, Japan [66]) or a Student’s *t*-test with a two-sided distribution. All experiments were performed more than twice with similar results unless otherwise indicated. Statistical significance was determined at *p* < 0.05.

## Data Availability

The original contributions presented in the study are included in the article/Supplemental, further inquiries can be directed to the corresponding author.

## Supplementary Materials

The following supporting information can be downloaded at https://www.mdpi.com/article/10.3390/ijms252212331/s1.

## Abbreviations

AP	alkaline phosphatase
APC	allophycocyanin
BBS	borate buffered saline
BCA	bicinchoninic acid
BSA	bovine serum albumin
CFA	complete Freund’s adjuvant
dLN	draining lymph node
ELISA	enzyme-linked immunosorbent assay
ELISPOT	enzyme-linked immuno spot
FCS	fetal calf serum
FITC	fluorescein isothiocyanate
GC	germinal center
KLH	keyhole limpet hemocyanin
MTT	3-(4,5-dimethylthiazol-2-yl)-2,5-diphenyl tetrazolium bromide
PBS	phosphate-buffered saline
PE	phycoerythrin
PECy7	phycoerythrin/cyanine7
PEI	polyethyleneimine
PI	propidium iodide
PP	Peyer’s patch
TD	T-dependent
Tfh	follicular helper T cell
Tfr	follicular regulatory T cells
TI	T-independent
TNF	tumor necrosis factor
TNFR	tumor necrosis factor receptor
TNP	2,4,6-trinitrophenol
TRAF	tumor necrosis factor receptor-associated factor
