# Supplementary material for: The Role of TNF Receptor-Associated Factor 5 in the Formation of Germinal Centers by B Cells During the Primary Phase of the Immune Response in Mice
Source: Int J Mol Sci. 2024 Nov 17;25(22):12331. doi: 10.3390/ijms252212331 (PMC11595067; doi:10.3390/ijms252212331)
Supplement: Supplementary file 1 [file ijms-25-12331-s001.zip › ijms-3323254-supplementary.pdf]

Figure S1

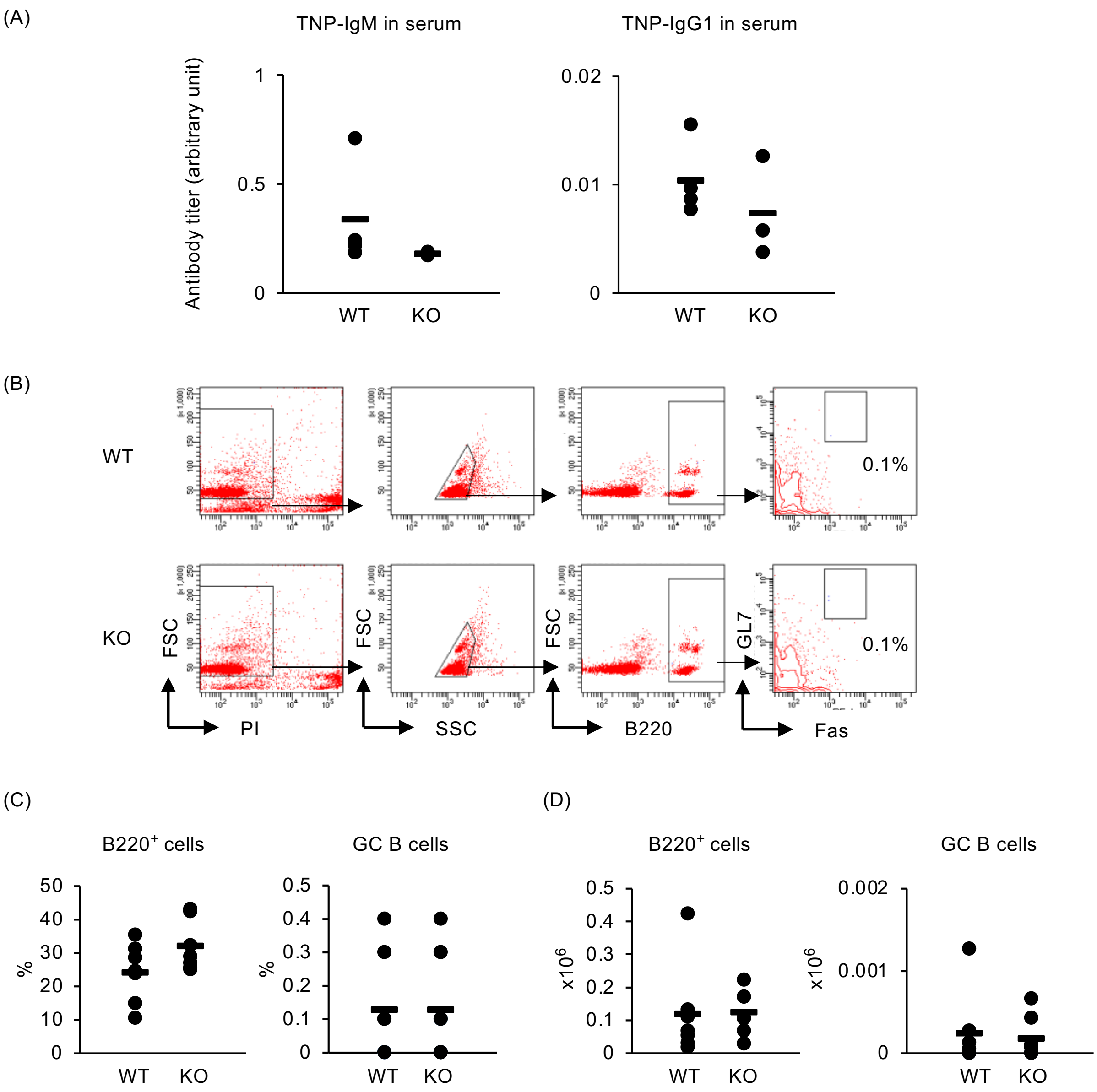

Figure S1. Anti-TNP antibodies and GC B cells in unimmunized *Traf5*$^{+/+}$ and *Traf5*$^{-/-}$ mice.

(A) Levels of TNP-specific IgM and IgG1 antibodies in the sera of unimmunized *Traf5*$^{+/+}$ (WT; n = 4) and *Traf5*$^{-/-}$ (KO; n = 3) mice. (B) Gating strategy for identifying propidium iodide (PI)-negative, Fas^{+}GL7^{+}B220^{+} GC B cells in the popliteal lymph node cells of WT and KO mice injected subcutaneously with PBS in the footpads as a control. (C, D) Percentages and counts of B220^{+} cells and GC B cells in the popliteal lymph node cells from unimmunized WT (n = 7) and KO (n = 7) mice. Bars represent the average values for individual mice. Statistical significance was determined using Student's *t*-test.

Figure S2

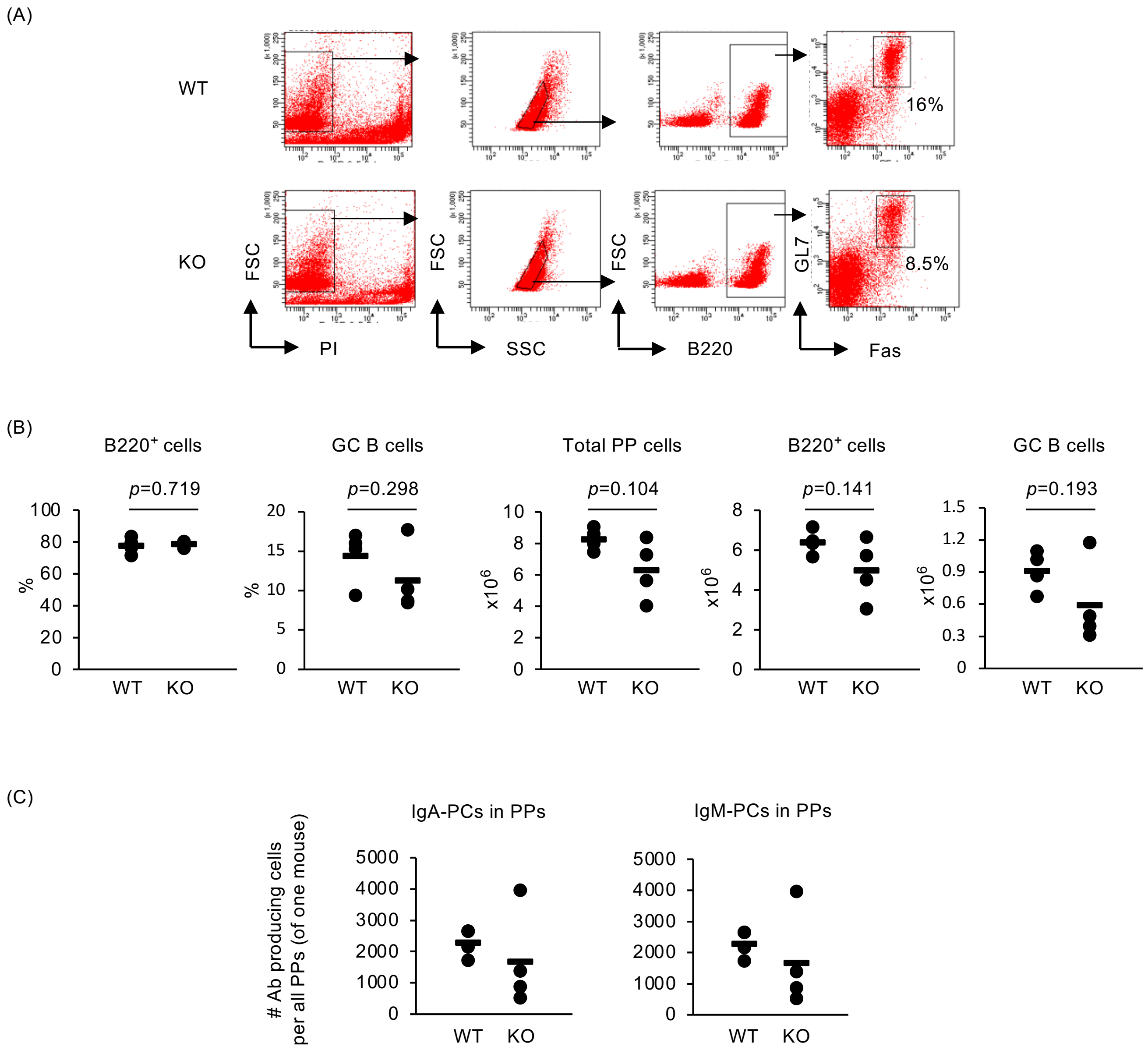

Figure S2. GC B cells from Peyer's patches in unimmunized *Traf5*$^{+/+}$ and *Traf5*$^{-/-}$ mice.

(A) Gating strategy for identifying PI-negative, Fas^{+}GL7^{+}B220^{+} GC B cells isolated from the Peyer's patches (PPs) of unimmunized WT and KO mice. (B) Percentages and counts of total cells from PPs, B220^{+} cells, and GC B cells in WT (n = 4) and KO (n = 4) mice. (C) Enumeration of IgA- and IgM-producing cells in PP cells from WT (n = 4) and KO (n = 4) mice, determined by ELISPOT. The numbers of IgA- and IgM-producing cells in all PPs from one mouse are shown. Bars represent the average values for individual mice. Statistical significance was determined using Student's *t*-test.

Figure S3

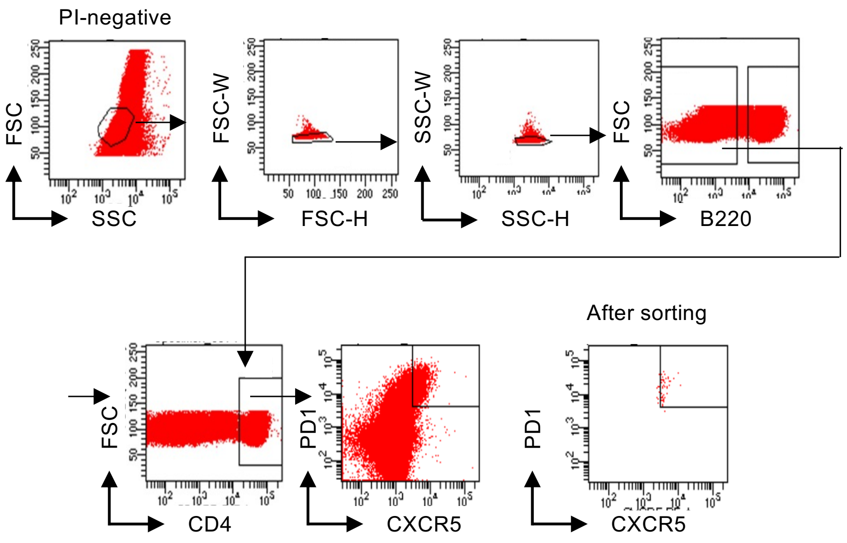

Figure S3. Gating strategy for sorted CXCR5$^+$PD-1$^+$CD4$^+$ T cells in TNP-KLH/CFA immunized *Traf5*$^{+/+}$ mice.

Representative gating strategy for sorting CXCR5$^+$PD-1$^+$CD4$^+$ T cells isolated from the popliteal LNs of TNP-KLH/CFA-immunized WT and KO mice (for detection of *Cd40lg* expression in Figure 3C).

Figure S4

Signal sequence—Fc (hinge, CH2 and CH3)—PA—MBL—CD40L—His

(EcoRI)—(START)—hIL-2Rγ signal sequence—(BamHI)—Fc(hinge-CH2-CH3)—PA—MBL—(KpnI)—CD40L—(AgeI)—His_6—(STOP)—(BglII)

GAATTCTCTAGAGATCCCTCGACCTCGAGATCCATCGGGCCCCCCCTCGAGGTCGACAGCAAGCGCCATGTTGAAGCCATCATTACCATTCACATCCCTCTTATTCCTGCAGCTGCCCC
TGCTGGGAGTGGGGCTGAACACGACAATTCTGACGCCCGGTAAGTATAGGATCCACTAGTTCTATCCTGCCTGGACGCATCCCGGCTATGCAGTCCCAGTCCAGGGCAGCAAGGCAGGC
CCCGTCTGCCTCTTCACCCGGAGGCCTCTGCCCGCCCCACTCATGCTCAGGGAGAGGGTCTTCTGGCTTTTTCCCCAGGCTCTGGGCAGGCACAGGCTAGGTGCCCCTAACCCAGGCCC
TGCACACAAAGGGGCAGGTGCTGGGCTCAGACCTGCCAAGAGCCATATCCGGGAGGACCCTGCCCCTGACCTAAGCCCACCCCAAAGGCCAAACTCTCCACTCCCTCAGCTCGGACACC
TTCTCTCCTCCCAGATTCCAGTAACTCCCAATCTTCTCTCTGCAGAGCCCAAATCTTGTGACAAAACTCACACATGCCCACCGTGCCCAGGTAAGCCAGCCCAGGCCTCGCCCTCCAGC
TCAAGGCGGGACAGGTGCCCTAGAGTAGCCTGCATCCAGGGACAGGCCCCAGCCGGGTGCTGACACGTCCACCTCCATCTCTTCCTCAGCACCTGAACTCCTGGGGGGACCGTCAGTCT
TCCTCTTCCCCCCAAAACCCAAGGACACCCTCATGATCTCCCGGACCCCTGAGGTCACATGCGTGGTGGTGGACGTGAGCCACGAAGACCCTGAGGTCAAGTTCAACTGGTACGTGGAC
GGCGTGGAGGTGCATAATGCCAAGACAAAGCCGCGGGAGGAGCAGTACAACAGCACGTACCGTGTGGTCAGCGTCCTCACCGTCCTGCACCAGGACTGGCTGAATGGCAAGGAGTACAA
GTGCAAGGTCTCCAACAAAGCCCTCCCAGCCCCCATCGAGAAAACCATCTCCAAAGCCAAAGGTGGGACCCGTGGGGTGCGAGGGCCACATGGACAGAGGCCGGCTCGGCCCACCCTCT
GCCCTGAGAGTGACCGCTGTACCAACCTCTGTCCCTACAGGGCAGCCCCGAGAACCACAGGTGTACACCCTGCCCCCATCCCGGGAGGAGATGACCAAGAACCAGGTCAGCCTGACCTG
CCTGGTCAAAGGCTTCTATCCCAGCGACATCGCCGTGGAGTGGGAGAGCAATGGGCAGCCGGAGAACAACTACAAGACCACGCCTCCCGTGCTGGACTCCGACGGCTCCTTCTTCCTCT
ATAGCAAGCTCACCGTGGACAAGAGCAGGTGGCAGCAGGGGAACGTCTTCTCATGCTCCGTGATGCATGAGGCTCTGCACAACCACTACACGCAGAAGAGCCTCTCCCTGTCCCCGGGT
AAAGGCGTTGCCATGCCAGGTGCCGAAGATGATGTGGTGTCATCAGGGTCACAAACCTGTGAGGACACCCTGAAGACTTGCTCTGTGATAGCCTGTGGCAGAGATGGAGAGATGGACC
CAAAGGGGAGAAGGGAGAACCAGGTCAAGGGCTCAGGGGCTTGCAGGGCCCTCCAGGGAAATTGGGGCCTCCAGGAAGTGTTGGAAGCCCTGGAAGTCCAGGACCAAAAGGCCAAAAGG
GGGACCATGGAGACAATAGAGCCATTGAGGAGAAGCTGGCAAATATGGAGGCAGAGATAAGGATCCTGAAATCAAAACTGCAGCTAACCAACAAGTTGCATGCCTTCTCAATGGGCAAA
AAGTCTGGGGGTACCAGAGGTGATGAGGATCCTCAAATTGCAGCACACGTTGTAAGCGAAGCCAACAGTAATGCAGCATCCGTTCTACAGTGGGCCAAGAAAGGATATTATACCATGAA
AAGCAACTTGGTAATGCTTGAAAATGGGAAACAGCTGACGGTTAAAAGAGAAGGACTCTATTATGTCTACACTCAAGTCACCTTCTGCTCTAATCGGGAGCCTTCGAGTCAACGCCCAT
TCATCGTCGGCCTCTGGCTGAAGCCCAGCAGTGGATCTGAGAGAATCTTACTCAAGGCGGCAAATACCCACAGTTCCTCCCAGCTTTGCGAGCAGCAGTCTGTTCACTTGGGCGGAGTG
TTTGAATTACAAGCTGGTGCTTCTGTGTTTGTCAACGTGACTGAAGCAAGCCAAGTGATCCACAGAGTTGGCTTCTCATCTTTTGGCTTACTCAAACTCACCGGTCATCATCACCATCA
CCATTGAAGATCT

Signal sequence–CD40–Fc (hinge, CH2 and CH3)–Flag–HA

(EcoRI)—(START)—hIL-2Rγ signal sequence—CD40—(BamHI)—Fc(hinge-CH2-CH3)—**Flag—HA**– (STOP)–(BglII)

GAATTCTCTAGAGATCCCTCGACCTCGAGATCCATCGGGCCCCCCCTCGAGGTCGACAGCAAGCGCCATGTTGAAGCCATCATTACCATTCACATCCCTCTTATTCCTGCAGCTGCCCC
TGCTGGGAGTGGGGCTGAACACGACAATTCTGACGCCCGTTACGTGCAGTGACAAACAGTACCTCCACGATGGCCAGTGCTGTGATTTGTGCCAGCCAGGAAGCCGACTGACAAGCCAC
TGCACAGCTCTTGAGAAGACCCAATGCCACCCATGTGACTCAGGCGAATTCTCAGCCCAGTGGAACAGGGAGATTCGCTGTCACCAGCACAGACACTGTGAACCCAATCAAGGGCTTCG
GGTTAAGAAGGAGGGCACCGCAGAATCAGACACTGTCTGTACCTGTAAGGAAGGACAACACTGCACCAGCAAGGATTGCGAGGCATGTGCTCAGCACACGCCCTGTATCCCTGGCTTTG
GAGTTATGGAGATGGCCACTGAGACCACTGATACCGTCTGTCATCCCTGCCCAGTCGGCTTCTTCTCCAATCAGTCATCACTTTTCGAAAAGTGTTATCCCTGGACAAGCTGTGAGGAT
AAGAACTTGGAGGTCCTACAGAAAGGAACGAGTCAGACTAATGTCATCTGTGGTTTAAAGTCCCGGATGCGAGGTAAGTATAGGATCCACTAGTTCTATCCTGCCTGGACGCATCCCGG
CTATGCAGTCCCAGTCCAGGGCAGCAAGGCAGGCCCCGTCTGCCTCTTCACCCGGAGGCCTCTGCCCGCCCCACTCATGCTCAGGGAGAGGGTCTTCTGGCTTTTTCCCCAGGCTCTGG
GCAGGCACAGGCTAGGTGCCCCTAACCCAGGCCCTGCACACAAAGGGGCAGGTGCTGGGCTCAGACCTGCCAAGAGCCATATCCGGGAGGACCCTGCCCCTGACCTAAGCCCACCCCAA
AGGCCAAACTCTCCACTCCCTCAGCTCGGACACCTTCTCTCCTCCCAGATTCCAGTAACTCCCAATCTTCTCTCTGCAGAGCCCAAATCTTGTGACAAAACTCACACATGCCCACCGTG
CCCAGGTAAGCCAGCCCAGGCCTCGCCCTCCAGCTCAAGGCGGGACAGGTGCCCTAGAGTAGCCTGCATCCAGGGACAGGCCCCAGCCGGGTGCTGACACGTCCACCTCCATCTCTTCC
TCAGCACCTGAACTCCTGGGGGGACCGTCAGTCTTCCTCTTCCCCCCAAAACCCAAGGACACCCTCATGATCTCCCGGACCCCTGAGGTCACATGCGTGGTGGTGGACGTGAGCCACGA
AGACCCTGAGGTCAAGTTCAACTGGTACGTGGACGGCGTGGAGGTGCATAATGCCAAGACAAAGCCGCGGGAGGAGCAGTACAACAGCACGTACCGTGTGGTCAGCGTCCTCACCGTCC
TGCACCAGGACTGGCTGAATGGCAAGGAGTACAAGTGCAAGGTCTCCAACAAAGCCCTCCCAGCCCCCATCGAGAAAACCATCTCCAAAGCCAAAGGTGGGACCCGTGGGGTGCGAGGG
CCACATGGACAGAGGCCGGCTCGGCCCACCCTCTGCCCTGAGAGTGACCGCTGTACCAACCTCTGTCCCTACAGGGCAGCCCCGAGAACCACAGGTGTACACCCTGCCCCCATCCCGGG
AGGAGATGACCAAGAACCAGGTCAGCCTGACCTGCCTGGTCAAAGGCTTCTATCCCAGCGACATCGCCGTGGAGTGGGAGAGCAATGGGCAGCCGGAGAACAACTACAAGACCACGCCT
CCCGTGCTGGACTCCGACGGCTCCTTCTTCCTCTATAGCAAGCTCACCGTGGACAAGAGCAGGTGGCAGCAGGGGAACGTCTTCTCATGCTCCGTGATGCATGAGGCTCTGCACAACCA
CTACACGCAGAAGAGCCTCTCCCTGTCCCCGGGTAAAGACTACAAGGATGACGATGACAAGCTCGATGGAGGATACCCATACGATGTTCCAGATTACGCTTGAAGATCT

Figure S4. Nucleotide sequences of Fc-CD40L and CD40-Fc proteins.

Figure S5

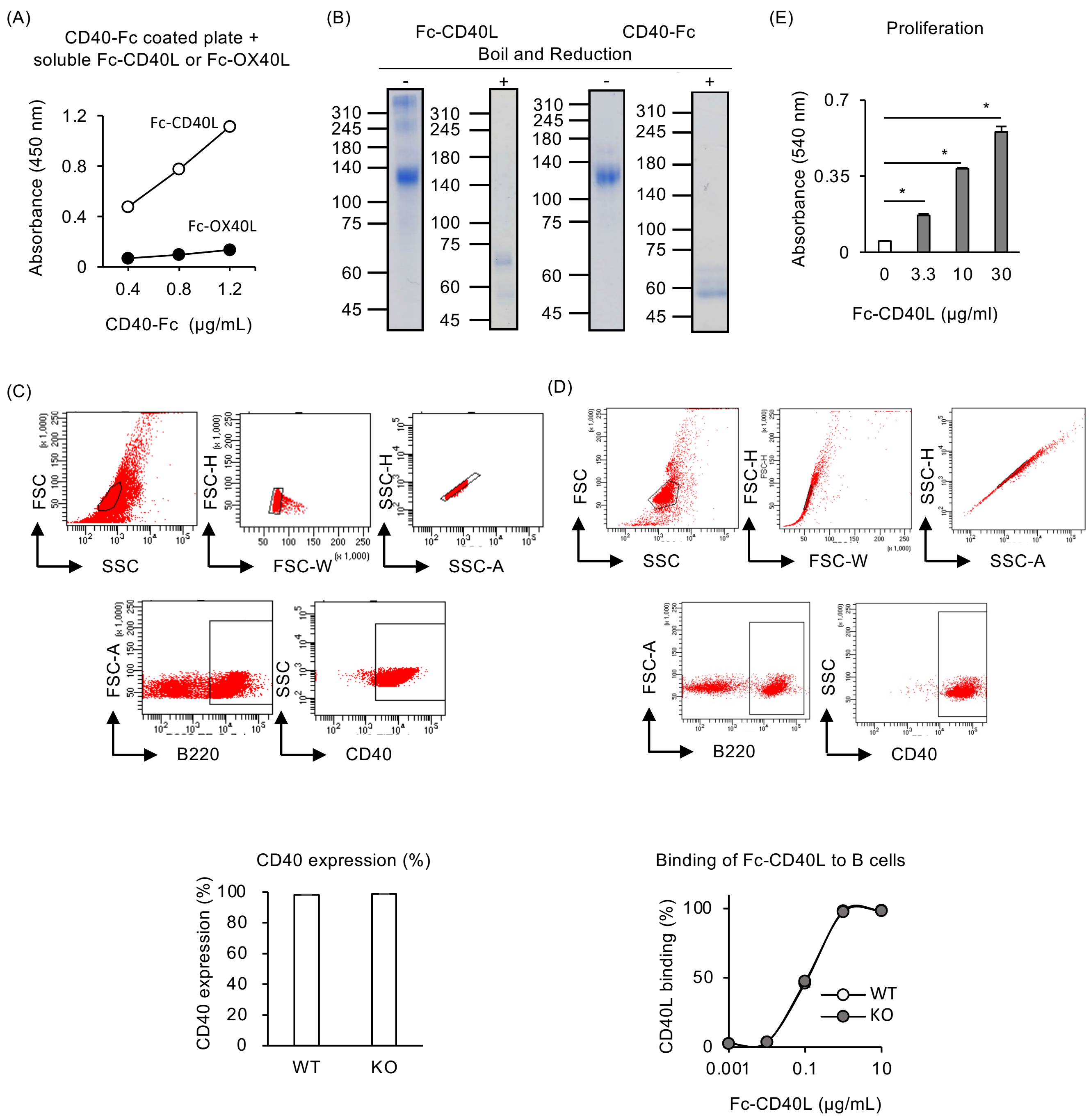

Figure S5. Functional analysis of the Fc-CD40L protein.
(A) Validation of specific interactions between Fc-CD40L and CD40-Fc by ELISA. ELISA plate wells were coated with the indicated concentrations of CD40-Fc, followed by the addition of 1 μg/mL of Fc-CD40L or Fc-OX40L. Fc-OX40L was used as a negative control. (B) SDS-PAGE (6%) analysis of Fc-CD40L and CD40-Fc under non-boiling/non-reducing (-) and boiling/reducing (+) conditions. (C, D) Expression of CD40 and binding activity of Fc-CD40L on B220^{+} B cells from WT and KO mice, evaluated by flow cytometry. (E) Proliferative responses of splenic naïve B cells stimulated with the indicated concentrations of Fc-CD40L and 10 ng/mL of IL-4, evaluated by MTT assay. Data represent a single experiment from two independent experiments showing similar results, presented as mean ± standard deviation (n = 3) (C, E). *$p < 0.05$ (E: one-way ANOVA with Tukey's post hoc test).

Figure S6

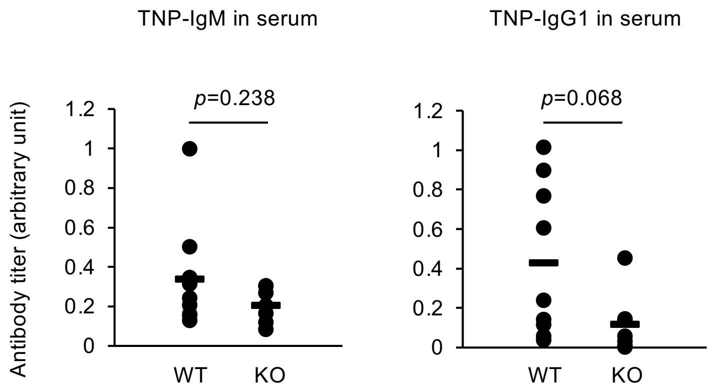

Figure S6. *Traf5*$^{-/-}$ mice primed with TNP-KLH and CD40L exhibit a trend toward decreased antigen-specific antibody responses.

Serum antibody responses on day 11 in WT (n = 9) and KO (n = 7) mice, as shown in Figure 6, were evaluated by ELISA. Bars represent mean values for individual mice. Statistical significance was assessed using Student's *t*-test.
